# The efficacy of vitamin D supplementation for irritable bowel syndrome: a systematic review with meta-analysis

**DOI:** 10.1186/s12937-022-00777-x

**Published:** 2022-05-05

**Authors:** Hangkai Huang, Linjie Lu, Yishu Chen, Yan Zeng, Chengfu Xu

**Affiliations:** 1 Department of Gastroenterology, the First Affiliated Hospital, Zhejiang University School of Medicine, No. 79 Qingchun Road, 310003 Hangzhou, China; 2 Department of Gastroenterology, Haining Branch of the First Affiliated Hospital, Zhejiang University School of Medicine, 314499 Haining, China

**Keywords:** Irritable bowel syndrome, Vitamin D, meta-analysis

## Abstract

**Background:**

Irritable bowel syndrome (IBS) is a chronic gastrointestinal disorder involving gut-brain interactions with limited effective treatment options. Vitamin D deficiency is commonly observed in patients with IBS, but whether vitamin D supplementation ameliorates IBS is controversial in randomized controlled trials. The present systematic review and meta-analysis explored the efficacy of vitamin D supplementation in patients with IBS.

**Methods:**

We performed a systematic search of potentially relevant publications from PubMed, EMBASE, the Cochrane Central Register of Controlled Studies and the Web of Science up until January 2022. We assessed the weighted mean difference (WMD) and 95% confidence interval (95% CI) of the IBS severity scoring system (IBS-SSS), IBS quality of life (IBS-QoL) and IBS total score (IBS-TS) before and after vitamin D supplementation intervention.

**Results:**

We included four randomized, placebo-controlled trials involving 335 participants. The differences in IBS-SSS score between participants in the intervention group and the placebo group increased after intervention (WMD: -55.55, 95% CI: -70.22 to -40.87, *I*^*2*^ = 53.7%, after intervention; WMD: -3.17, 95% CI: -18.15 to 11.81, *I*^*2*^ = 0.0%, before intervention). Participants receiving vitamin D supplementation showed greater improvement in IBS-SSS after intervention than participants receiving placebo treatment (WMD: -84.21, 95% CI: -111.38 to -57.05, *I*^*2*^ = 73.2%; WMD: -28.29, 95% CI: -49.95 to -6.62, *I*^*2*^ = 46.6%, respectively). Vitamin D supplementation was also superior to placebo in IBS-QoL improvement (WMD: 14.98, 95% CI: 12.06 to 17.90, *I*^*2*^ = 0.0%; WMD: 6.55, 95% CI: -2.23 to 15.33, *I*^*2*^ = 82.7%, respectively). Sensitivity analyses revealed an unstable pooled effect on IBS-TS in participants receiving vitamin D supplementation. Therefore, we did not evaluate the efficacy of vitamin D intervention in IBS-TS.

**Conclusions:**

This systematic review and meta-analysis suggested that vitamin D supplementation was superior to placebo for IBS treatment.

**Supplementary information:**

The online version contains supplementary material available at 10.1186/s12937-022-00777-x.

## Introduction

Irritable bowel syndrome (IBS) is a chronic gastrointestinal disorder involving gut-brain interactions that features recurrent abdominal pain and a changed frequency or form of stool [1]. According to the Rome III criteria, the global prevalence of IBS is 9.2%, and regional prevalence ranges from 0.4 to 29.2% [2]. When the Rome IV criteria, a more restrictive version revised in 2016 [3], was adopted, the global prevalence of IBS was 3.8% [2]. IBS exerts a negative influence on work productivity and quality of life [1]. Patients suffering from IBS may take more time off work and struggle to perform well at work [4], and the extent of work impairment is related to the severity of symptoms [5]. Numerous first-line and second-line therapies for IBS have been established [6]. The bulk of current therapies focus on the most predominant symptoms [7]. However, a personalized and precise treatment for IBS should focus on the underlying pathophysiology rather than predominant symptoms [8]. Novel approaches with limited adverse effects targeting the underlying mechanisms of IBS are needed.

Vitamin D is a fat-soluble vitamin with hormone-like properties that regulates calcium and phosphate homeostasis [9]. Vitamin D deficiency is gradually becoming a global health burden, with a prevalence of 28.9% in the United States [10] and 34.2% in Africa [11]. Several observational studies reported that patients with IBS had a higher prevalence of vitamin D deficiency than controls [12], and vitamin D status was inversely related to IBS [13]. Interventional studies demonstrated that vitamin D supplementation improved depression and anxiety [14, 15], which are common psychological comorbidities in patients with IBS [1]. In addition to the traditional role of vitamin D in skeletal diseases, mounting studies have provided new insights into its anti-inflammatory effect [16]. Low-grade intestinal epithelial inflammation also plays a role in the pathogenesis of IBS [17].

New effective treatments for IBS are needed, and vitamin D may target different aspects of IBS pathophysiology. Therefore, we performed a meta-analysis and systematic review to examine whether vitamin D supplementation effectively improved the symptoms and the quality of life in patients with IBS.

## Method

### Search strategy

We performed a systematic search of potentially relevant publications from PubMed, EMBASE, the Cochrane Central Register of Controlled Studies and the Web of Science until January 2022. We also searched ClinicalTrials.gov for eligible articles and unpublished trials. No language or age restrictions were imposed. We combined subject and free-text terms of IBS with vitamin D and randomized controlled trials using the set operator AND as our search strategy. To identify more eligible articles, we scanned the references of the reviewed articles. Double-blinded, placebo-controlled, randomized studies evaluating the effectiveness of vitamin D supplementation in the treatment of IBS were included. Vitamin D dose and treatment duration were not pre-specified. We excluded duplicated studies and studies with irrelevant subjects. The complete search strategy is listed in Supplementary Table S1.

### Data extraction

Two investigators separately extracted the data needed, including author, publication year, country, study design, number of patients in each group, diagnostic criteria of IBS, IBS subtypes, vitamin D dose, treatment duration and primary and secondary outcomes. The primary outcome was measured using the IBS-severity scoring system (IBS-SSS), which contains 5 items that are scored from 0 to 100 [18]. Higher scores represent more severe symptoms. A decline greater than 50 is considered a significant improvement [18]. The secondary outcome was measured using IBS-quality of life (IBS-QoL) and IBS-total score (IBS-TS), which range from 0 to 100 [19]. In contrast to IBS-SSS scores, lower scores of IBS-QoL and IBS-TS represent poorer outcomes. An increase of 10 points or more in IBS-QoL was viewed as a significant improvement [20]. All data were extracted using intention-to-treat analyses, and discrepancies were resolved by discussion.

### Assessment of risk of bias

The risk of bias in each included study was assessed using the Cochrane Collaboration’s tool [21], which considered the following factors: adequate randomization of sequence generation, methods used to conceal allocation, blinding of all measures to participants, eligibility of researchers and outcome assessors, completeness of each outcome, and absence of selective reporting.

### Assessment of quality of evidence

Two investigators separately assessed the quality of evidence of IBS-SSS, IBS-QoL and IBS-TS using the Grading of Recommendations Assessment, Development and Evaluation (GRADE) [22].

### Statistical analysis

We analyzed the weighted mean difference (WMD) of IBS-SSS, IBS-QoL and IBS-TS before and after vitamin D supplementation or placebo treatment. Chi-squared and *I*^*2*^ statistics were used to assess heterogeneity, and *I*^*2*^ < 25% and *P* > 0.01 were considered insignificant heterogeneity. Data of studies with significant heterogeneity were analyzed using the random-effect model as described by DerSimonian and Laird. The influence of each study on the pooled effect was measured using sensitivity analyses. Publication bias was quantified using Begg’s test and Egger’s test. Two investigators performed all analyses using STATA 15 (Stata Corporation, College Station, TX) separately.

## Result

### Search results

**Fig. 1:**
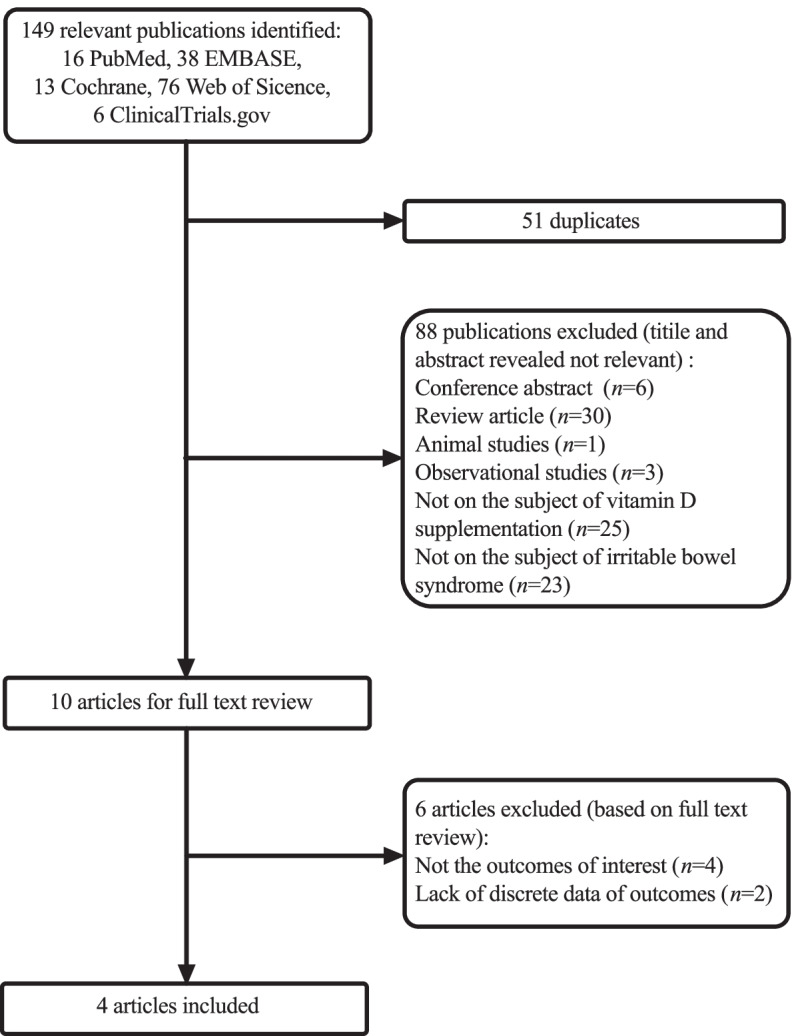
Flow diagram of study selection in this systematic review and meta-analysis

We initially identified 149 publications, of which 51 duplicates and 88 records with irrelevant subjects or an inapplicable study type were excluded. Of the 10 remaining articles, 6 studies were excluded after full-text review due to incomplete outcomes or unrelated measurements. Four studies were ultimately included in this meta-analysis (Fig. 1).

Overall, 169 participants receiving vitamin D supplementation and 166 receiving placebos were included using intention-to-treat analyses. As shown in Table 1, three trials, but one diagnosed IBS using the Rome III criteria, because these three trials started before 2016 when the Rome III criteria were revised to a Rome IV [3]. Three studies included all subtypes of IBS, and one study included only IBS-diarrhea. The combination of vitamin D dose and treatment duration varied across studies. A dose of 50,000 IU of vitamin D was provided for the experimental groups fortnightly in two trials, which differed in treatment duration, of 6 weeks or 6 months. A vitamin D dose of 50,000 IU or 2000 IU was provided weekly or daily, respectively, in the other two trials, and the duration of therapy was 9 weeks or 6 months, respectively. There was no report on any adverse effect of vitamin D supplementation in the included trials.

**Table 1:** Characteristics of randomized controlled trials of vitamin D supplementation on IBS

Study	Country	Study design	IBS criteria	Case	Control	Vitamin D dose	Treatment duration	Primary outcomes	Secondary outcomes
				(*n*)	(*n*)				
Abbasnezhad 2016 [23]	Iran	Double-blind	IBS-C 34.1%, IBS-D 25.9%, IBS-A 40%	44	41	50000 IU VD_3_ fortnightly	6 months	IBS-SSS	IBS-QoL, the measurement of GI symptoms
Khalighi Sikaroudi 2020 [24]	Iran	Double-blind	IBS-D	44	44	50000 IU VD_3_ weekly	9 weeks	IBS-SSS	IL-6, CRH
El Amrousy 2018 [25]	Egypt	Double-blind	IBS-C 61.8%, IBS-D 18.6%, IBS-M 10.8%, IBS-U 8.9%	56	56	2000 IU VD_3_ daily	6 months	IBS-SSS	IBS-QoL, IBS-TS
Jalili 2016 [26]	Iran	Double-blind	all subtypes (the proportion of each subtype was unclear)	25	25	50000 IU VD_3_ fortnightly	6 weeks	IBS-SSS	IBS-TS

*IBS* irritable bowel syndrome, *IBS-C, -D, -A, -M, -U* IBS with predominant constipation, predominant diarrhea, predominant alternating bowel habit, mixed and unsubtyped, *IBS-QoL* IBS-quality of life, *IBS-SSS* IBS-severity scoring system, *IBS-TS* IBS-total score, *IU* international unit, *VD*_*3*_ vitamin D_3_

The risks of bias of the included studies are summarized in Table 2. The risks of random sequence generation and blinding to the outcome assessment in Jalili et al. [26] were not clear. The other risks considered in these studies were all low. Table 3 showed that the GRADE quality of IBS-SSS and IBS-TS were very low and the quality of IBS-QoL was low.

**Table 2:** Risk of bias of studies included in this meta-analysis

Study	Random sequence generation	Allocation concealment	Blinding of participants, personnel	Blinding of outcome assessment	Incomplete outcome data	Selective reporting	Other sources of bias
Abbasnezhad 2016 [23]	Low risk; computer-generated blocked randomization list with a block size of 6	Low risk; quote "all the participants, researchers, and the physician were blind to the allocations using the random codes"	Low risk, double-blind	Low risk, double-blind	Low risk, quote "All analyses were done on the intention-to-treat population"	Low risk, all prespecified outcomes were reported	Low risk
Khalighi Sikaroudi 2020 [24]	Low risk; randomization was performed by using the online site www. sealedenvelope.com	Low risk; quote "For performing the concealment in the randomization process, dedicated codes were used on the pharmaceutical sheets, which were generated by the software."	Low risk, double-blind	Low risk, double-blind	Low risk, intention-to-treat analysis	Low risk, all prespecified outcomes were reported	Low risk
El Amrousy 2018 [25]	Low risk; quote "computer-generated random numbers using a random block size of 6"	Low risk; quote "Allocation concealment was done by sequentially numbered sealed opaque envelopes."	Low risk; participants and treating staff were blinded to the treatment group.	Low risk; all outcome assessors were blinded to the treatment group	Low risk, complete outcome	Low risk, all prespecified outcomes were reported	Low risk
Jalili 2016 [26]	Unclear; the author reported that participants were randomly assigned to different groups but did not mention how the random sequence was generated	Low risk; randomization was provided in sealed opaque envelopes with successive numbers.	Low risk; all participants and researchers were blinded to the treatment group.	Unclear; insufficient information	Low risk, intention-to-treat analysis	Low risk, all prespecified outcomes were reported	Low risk

**table 3:** GRADE quality of evidence of vitamin D versus placebo on IBS

Outcome	Risk of bias	Inconsistency	Indirectness	Imprecision	Publication bias	Certainty
IBS-SSS	Not serious	Serious	Not serious	Serious	Serious	⨁○○○ Very low
IBS-QoL	Serious	Serious	Not serious	Serious	Serious	⨁⨁○○ Low
IBS-TS	Not serious	Serious	Not serious	Serious	Serious	⨁○○○ Very low

*IBS* irritable bowel syndrome, *IBS-QoL *IBS-quality of life, *IBS-SSS *IBS-severity scoring system, *IBS-TS *IBS-total score

## Differences in IBS-SSS, IBS-QoL and IBS-TS before and after intervention

**Fig. 2:**
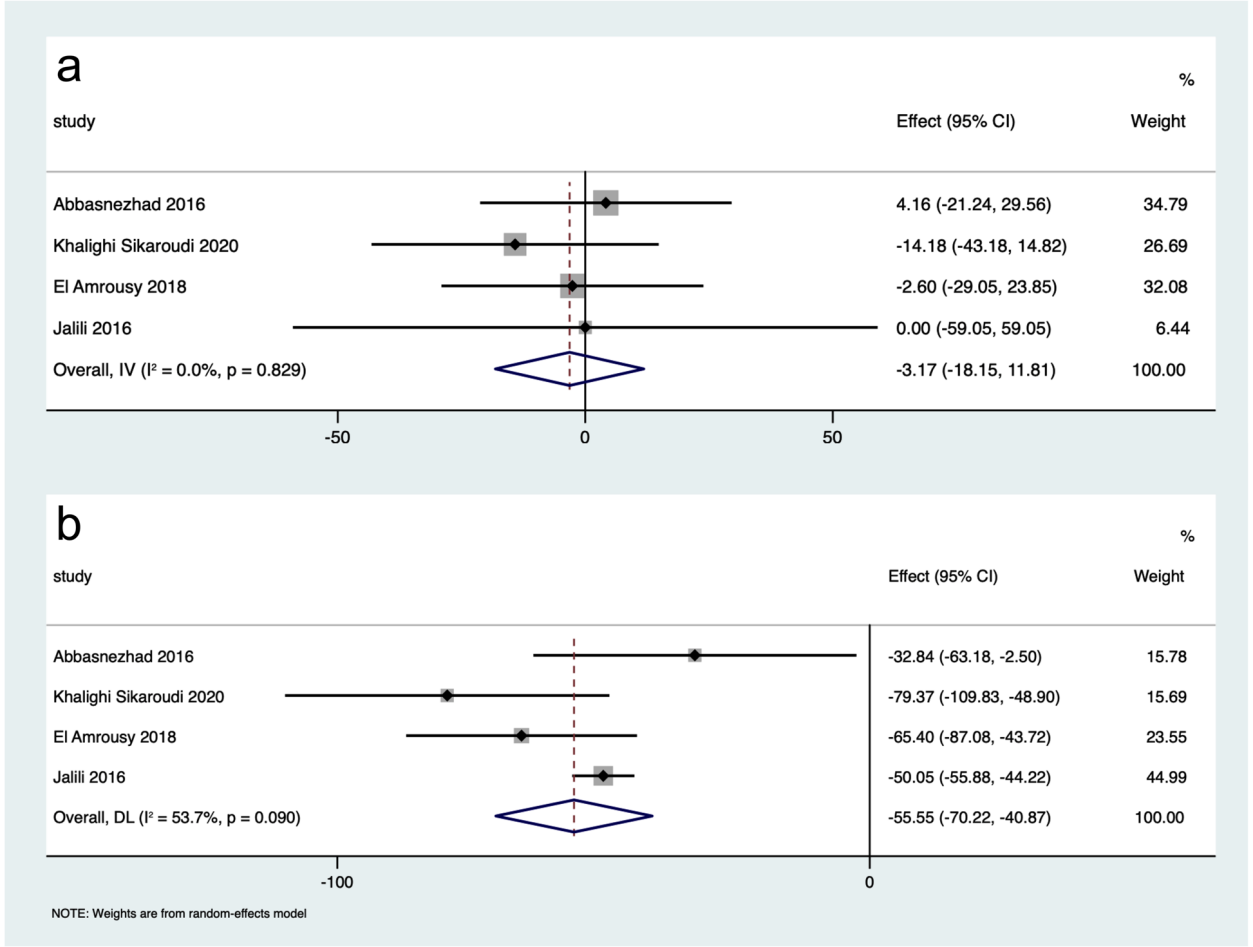
Forest plot of the differences in IBS-SSS scores between participants in the intervention group and placebo group before and after intervention. **a** The differences in IBS-SSS scores between participants in the intervention group and placebo group before intervention; **b** the differences in IBS-SSS scores between participants in the intervention group and placebo group after intervention

**Fig. 3:**
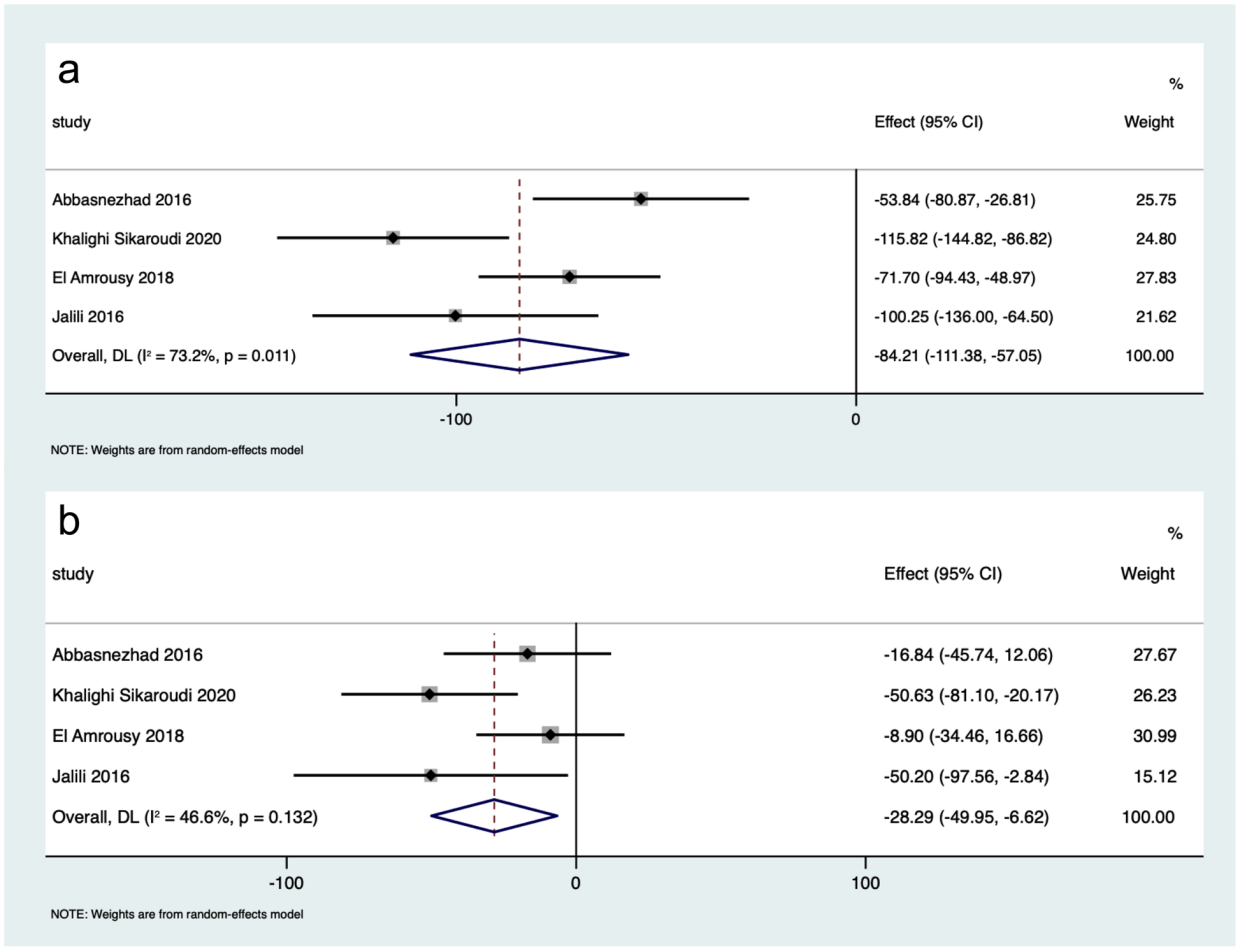
Forest plot of the improvement of IBS-SSS after intervention in participants receiving vitamin D supplementation and receiving placebo. **a** The improvement of IBS-SSS in participants receiving vitamin D supplementation; **b** the improvement of IBS-SSS in participants receiving placebo

**Fig. 4:**
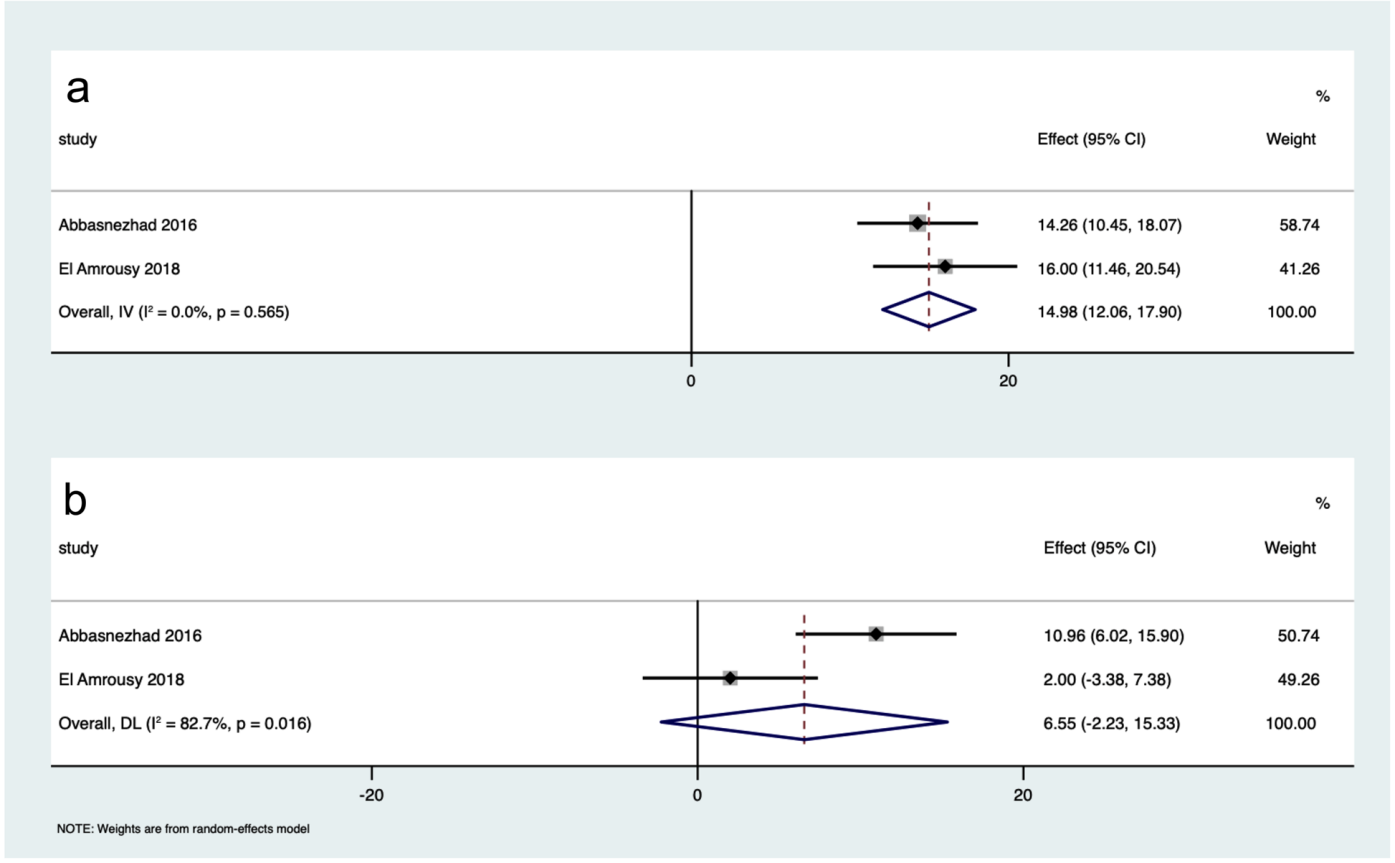
Forest plot of the improvement of IBS-QoL after intervention in participants receiving vitamin D supplementation and receiving placebo. **a** The improvement of IBS-QoL in participants receiving vitamin D supplementation; **b** the improvement of IBS-QoL in participants receiving placebo

The differences in IBS-SSS scores between participants in the intervention group and the placebo group were greater after intervention than before intervention (WMD: -55.55, 95% CI: -70.22 to -40.87, *I*^*2*^ = 53.7%; WMD: -3.17, 95% CI: -18.15 to 11.81, *I*^*2*^ = 0.0%, respectively) (Fig. 2). Participants receiving vitamin D supplementation showed greater improvement of IBS-SSS than controls (WMD: -84.21, 95% CI: -111.38 to -57.05, *I*^*2*^ = 73.2%; WMD: -28.29, 95% CI: -49.95 to -6.62, *I*^*2*^ = 46.6%, respectively) (Fig. 3). Vitamin D supplementation was superior to placebo in improving IBS-QoL (WMD: 14.98, 95% CI: 12.06 to 17.90, *I*^*2*^ = 0.0%; WMD: 6.55, 95% CI: -2.23 to 15.33, *I*^*2*^ = 82.7%, respectively) (Fig. 4). These results suggested that vitamin D supplementation contributed to a significant improvement in the symptoms and the quality of life in patients with IBS.

## Sensitivity analyses and assessment of publication bias

Sensitivity analyses for each of the aforementioned studies showed that the pooled effect remained stable when we omitted each of the studies (Supplementary Figures S1 to S6). However, when we performed sensitivity analyses on the improvement in IBS-TS in participants receiving vitamin D supplementation, the pooled effect was unstable. Therefore, we excluded IBS-TS from our evaluation tools.

We were unable to assess the publication bias due to the limited number of studies included, according to the Cochrane handbook [27].

## Discussion

The present systematic review and meta-analysis assessed the efficacy of vitamin D supplementation in improving IBS. We included 4 randomized controlled trials involving 335 participants and found that vitamin D supplementation contributed to improvement of symptoms and quality of life in patients with IBS.

Numerous first-line and second-line therapies for IBS have been established, and emerging treatments were efficacious in randomized controlled trials [6]. Most of these therapies focused on the most predominant symptoms [7]. For example, the first-line therapy for IBS patients with predominant constipation is laxatives and soluble fibers, and the second-line therapy is intestinal secretagogues and 5-hydroxytryptamine_4_ agonists. Emerging treatments include elobixibat, mizagliflozin and DRAinh-A250 [28]. Abundant treatments for IBS from different aspects, including dietary modification [7], pharmacological [29] and psychological therapies [30], were reported, but how many patients benefit from these treatments and whether the accompanying adverse effects are tolerable remain controversial [28]. Ford et al. [28] summarized that most randomized controlled trials that assessed the efficacies of these treatments had evident methodological limitations, including high risks of research bias, limited coverage of populations, heterogeneity between studies and possible publication bias. For example, the 5-hydroxytryptamine_3_ antagonist alosetron, which targets IBS with predominant diarrhea, was used restrictively due to its side effects of severe constipation and ischemic colitis [31]. A safer 5-hydroxytryptamine_3_ antagonist, ramosetron, caused constipation more often than placebo [28]. A personalized and precise treatment for IBS should focus on the underlying pathophysiology rather than predominant symptoms [8]. Therefore, other approaches with limited adverse effects targeting the underlying mechanisms are needed.

We found that vitamin D supplementation showed protective effects on the symptoms and quality of life in patients with IBS in this systematic review and meta-analysis. Vitamin D supplementation may be a promising therapy for the treatment of IBS. Our findings provide significant implications for clinical practice and research. Vitamin D supplementation may be a promising therapy for IBS and target different aspects of IBS pathophysiology. Many first-line therapies targeting the most predominant symptoms of IBS, such as loperamide for diarrhea [32, 33], soluble fibers for constipation [33, 34] and antispasmodic drugs for abdominal pain [35], have beneficial effects on certain symptoms of IBS but contribute little to other symptoms. The symptoms of IBS may shift over time [36, 37]. Therefore, the development of a more individualized and precise treatment strategy for IBS should focus on the underlying pathophysiology of IBS rather than its predominant symptoms [8]. Vitamin D supplementation may meet this goal. Our analyses have fewer methodological limitations than the previous analyses. The Rome criteria for IBS diagnosis was revised from I to IV over the past two decades [38]., and the randomized controlled trials on IBS performed during this time used different versions of the Rome criteria [39, 40]. For example, most randomized controlled trials on alosetron and early trials on ramosetron used Rome I or II [39], but later trials on ramosetron used Rome III [40]. The heterogeneity of participant selection in these trials cannot be ignored, and their pooled effects should be taken more seriously [29].

Notably, most of the participants included in this study were vitamin D deficient or insufficient. The participants enrolled in Jalili et al. [26] and Khalighi et al. [24] had serum vitamin D levels lower than 30 ng/mL, and El Amrousy et al. [25] enrolled participants with serum vitamin D levels lower than 20 ng/mL. In the study conducted by Abbasnezhad et al. [23], the proportions of participants who had serum vitamin D < 20 and < 30 ng/mL were 65.9% and 84.1%, respectively. Abbasnezhad et al. [23] showed that the effects of vitamin D on improving IBS-SSS and IBS-QoL were more significant in participants with vitamin D insufficiency than in participants without insufficiency. Patients with vitamin D insufficiency showed greater improvement in glycemic control after vitamin D supplementation than patients without insufficiency [41, 42]. Further studies are needed to explore the dose and duration of vitamin D therapy that are suitable for different ages, races, sexes and vitamin D statuses.

Notably, participants receiving placebo treatment showed improved IBS-SSS compared with baseline, which indicates placebo effects. Placebo effects may corrupt the efficacy of novel therapies [43]. Previous meta-analyses reported that placebo response rates were 20 − 40% in clinical trials in patients with IBS [44]. Studies with longer trial durations, shorter run-in periods and performed earlier were associated with an increased placebo response rate [45]. Although we observed placebo effects in this meta-analysis, the improvement in IBS-SSS was more significant in participants receiving vitamin D supplementation than the controls.

The precise mechanism of the effectiveness of supplementary vitamin D in improving IBS requires further elucidation. One possible explanation is that vitamin D ameliorates inflammation and the psychological-psychiatric state. Several studies demonstrated that chronic low-grade intestinal mucosal inflammation was a crucial part of the pathogenesis of IBS [17]. Increased release of pro-inflammatory cytokines, including tumor necrosis factor-α, interleukins-6 and − 1β, was found in peripheral mononuclear cells [46] and close to enteric nerve fibers in the intestinal mucosa of patients with IBS [47, 48]. Researchers also observed activated humoral immune responses in peripheral circulation [49] and jejunal mucosa [50] in patients with IBS, characterized by increased proliferation and activation of B lymphocytes [50]. The aforementioned elevated concentrations of cytokines and activated humoral immune responses were positively associated with the severity and frequency of abdominal pain, changed bowel habits [50], and even depression and anxiety [46, 51]. The integrity of the intestinal epithelial barrier is impaired in patients with IBS, presented as changes in tight junction proteins [52]. However, experimental studies found that vitamin D contributed to maintaining the integrity of the intestinal epithelial barrier by inhibiting apoptosis of epithelial cells [53] and regulating tight junction proteins [54]. Vitamin D also exhibited anti-inflammatory effects by suppressing T helper 1 and T helper 17 cells [55, 56] and downregulating the interleukin-23 receptor pathway in innate lymphoid cells [57].

Psychological conditions, such as anxiety and depression, often coexist with IBS [58]. Notably, psychological conditions may be a consequence or predecessor of IBS [59]. For example, patients with more severe anxiety but without IBS at baseline were more likely to develop IBS after a 1-year [60] and 12-year follow-up [61]. Patients with IBS but without psychological comorbidities at baseline had a higher risk of developing anxiety and depression [60, 61]. Therefore, bidirectional communication exists between the central and enteric nervous systems, named gut-brain interactions [61]. The central nervous system influences visceral sensitivity and bowel motility, which are closely related to the typical symptoms of IBS, such as abdominal pain and changed bowel habits. The central nervous system also receives feedback from the bowel and affects psychological conditions [61]. A low level of serum vitamin D is a significant predictor of depression [62], and supplementary vitamin D ameliorates symptoms of anxiety and depression [14, 15], by reducing the elevated concentration of Ca^2+^ in inhibitory neurons [63]. Overall, vitamin D may exert beneficial effects on IBS via multiple mechanisms that are crucial to the pathogenesis of IBS.

There are several limitations in the present systematic review and meta-analysis. First, only four studies were included. Due to this limited number, publication bias was not evaluated. Second, three of the four included studies were from Iran, and the other was from Egypt. We also found several relevant studies performed in other countries, which were regretfully terminated due to low enrollment. For example, one trial in the United States was terminated because only 7 participants were ultimately enrolled. The effectiveness of supplementary vitamin D in treating IBS must be further elucidated in other populations. Third, the risk of adequate random sequence generation and information on blinding in the study design were unclear in Jalili et al. [26].

## Conclusions

In summary, our results suggest that vitamin D supplementation effectively improves the symptoms and the quality of life in patients with IBS. Vitamin D is inexpensive and safe and may be a useful and practical approach for IBS treatment.

## Supplementary Information

**Additional file 1.**

## Data Availability

The data of the present study will be available for the corresponding author.
