# Supplementary material for: The efficacy of vitamin D supplementation for irritable bowel syndrome: a systematic review with meta-analysis
Source: Nutr J. 2022 May 5;21:24. doi: 10.1186/s12937-022-00777-x (PMC9069731; doi:10.1186/s12937-022-00777-x)
Supplement: Supplementary file 1 — Additional file 1. [file 12937_2022_777_MOESM1_ESM.docx]

**The efficacy of vitamin D supplementation for irritable bowel syndrome: a systematic review with meta-analysis**

**(VD supplementation and IBS)**

Hangkai Huang^1,†^, Linjie Lu^1,2,†^, Yishu Chen^1,†^, Yan Zeng^1^, Chengfu Xu^1,2,*^

^1^ Department of Gastroenterology, the First Affiliated Hospital, Zhejiang University School of Medicine, Hangzhou 310003, China

^2^ Department of Gastroenterology, Haining Branch of the First Affiliated Hospital, Zhejiang University School of Medicine, Haining 314499, China

^†^ These authors contributed equally to this work.

^*^**Correspondence:**

Chengfu Xu, M.D.

Department of Gastroenterology,

The First Affiliated Hospital, Zhejiang University School of Medicine,

No. 79 Qingchun Road, Hangzhou 310003, China.

Phone: 0086-571-87236532

E-mail: [xiaofu@zju.edu.cn](mailto:xiaofu@zju.edu.cn)

**Table S1. Search strategies**

| Database | Search strategy |
| --- | --- |
| PubMed | ((“Vitamin D”[Mesh]) OR Cholecalciferol OR Hydroxycholecalciferol OR Calcifediol OR Dihydroxycholecalciferol OR Dihydroxyvitamin d OR Dihydrotachysterol OR ergocalciferol OR alphacalcidol OR calcitriol) AND ((randomized controlled trial) OR (controlled clinical trial) OR randomized OR placebo OR trial) AND ((“Irritable Bowel Syndrome”[Mesh]) OR (Irritable colon) OR (Irritable bowel) OR (spastic colon)) |
| EMBASE | (‘vitamin d’/exp OR ‘vitamin d’ OR ergocalciferol OR colecalciferol OR calcitriol OR alfacalcidol OR calcifediol OR hydroxycolecalciferol OR dihydroxycolecalciferol) AND (‘irritable colon’/exp OR ‘irritable colon’) AND (‘randomized controlled trial’/exp OR ‘randomized controlled trial’ OR ‘single blind procedure’ OR ‘double blind procedure’ OR ‘crossover procedure’) |
| Cochrane central register of controlled study | ([Vitamin D] explode all trees OR (Cholecalciferol):ti,ab,kw OR (Calcifediol):ti,ab,kw OR (calcitriol):ti,ab,kw OR (alphacalcidol):ti,ab,kw OR (ergocalciferol):ti,ab,kw OR (Dihydrotachysterol):ti,ab,kw OR (Dihydroxycholecalciferol):ti,ab,kw OR (“Dihydroxyvitamin d”):ti,ab,kw OR (Hydroxycholecalciferol):ti,ab,kw) AND ([Irritable Bowel Syndrome] explode all trees OR (“irritable colon”):ti,ab,kw OR (“Irritable bowel”):ti,ab,kw OR (“spastic colon”):ti,ab,kw) |
| Web of Science | ((TS=(Vitamin D) OR TS=(cholecalciferol) OR TS=(ergocalciferol) OR TS=(alphacalcidol) OR TS=(calcitriol) OR TS=(Hydroxycholecalciferol) OR TS=(Dihydroxycholecalciferol) OR TS=(Dihydrotachysterol) OR TS=(Dihydroxyvitamin d)) AND ((TS=(irritable bowel syndrome) OR TS=(irritable colon) OR TS=(irritable bowel) OR TS=(spastic colon)) AND ((TS=(placebo)) OR TS=(random) OR TS=(clinical trial) OR TS=(double blind) OR TS=(single blind) OR TS=(randomized clinical trial)) |
| ClinicalTrials.gov | Interventional Studies \| Irritable Bowel Syndrome \| Vitamin D |

**Table S2. PRISMA 2009 Checklist ^1^**

| **Section/topic** | **Item**  **#** | **Checklist item** | **Reported on page #** |
| --- | --- | --- | --- |
| **Title** |  |  |  |
| Title | 1 | Identify the report as a systematic review, meta-analysis, or both | 1 |
| **Abstract** |  |  |  |
| Structured summary | 2 | Provide a structured summary including, as applicable, background, objectives, data sources, study eligibility criteria, participants, interventions, study appraisal and synthesis methods, results, limitations, conclusions and implications of key findings, systematic review registration number | 3 |
| **Introduction** |  |  |  |
| Rationale | 3 | Describe the rationale for the review in the context of what is already known | 4 |
| Objectives | 4 | Provide an explicit statement of questions being addressed with reference to participants, interventions, comparisons, outcomes, and study design (PICOS) | 4 |
| **Methods** |  |  |  |
| Protocol and registration | 5 | Indicate if a review protocol exists, if and where it can be accessed (such as web address), and, if available, provide registration information including registration number | 6 |
| Eligibility criteria | 6 | Specify study characteristics (such as PICOS, length of follow-up) and report characteristics (such as years considered, language, publication status) used as criteria for eligibility, giving rationale | 6 |
| Information sources | 7 | Describe all information sources (such as databases with dates of coverage, contact with study authors to identify additional studies) in the search and date last searched | 6 |
| Search | 8 | Present full electronic search strategy for at least one database, including any limits used, such that it could be repeated | 6 |
| Study selection | 9 | State the process for selecting studies (that is, screening, eligibility, included in systematic review, and, if applicable, included in the meta-analysis) | 6 |
| Data collection process | 10 | Describe method of data extraction from reports (such as piloted forms, independently, in duplicate) and any processes for obtaining and confirming data from investigators | 6 |
| Data items | 11 | List and define all variables for which data were sought (such as PICOS, funding sources) and any assumptions and simplifications made | 6 |
| Risk of bias in individual studies | 12 | Describe methods used for assessing risk of bias of individual studies (including specification of whether this was done at the study or outcome level), and how this information is to be used in any data synthesis | No |
| Summary measures | 13 | State the principal summary measures (such as risk ratio, difference in means) | 6 |
| Synthesis of results | 14 | Describe the methods of handling data and combining results of studies, if done, including measures of consistency (such as I2) for each meta-analysis | 6 |
| Risk of bias across studies | 15 | Specify any assessment of risk of bias that may affect the cumulative evidence (such as publication bias, selective reporting within studies) | 6 |
| Additional analyses | 16 | Describe methods of additional analyses (such as sensitivity or subgroup analyses, meta-regression), if done, indicating which were pre-specified | 6 |
| **Results** |  |  |  |
| Study selection | 17 | Give numbers of studies screened, assessed for eligibility, and included in the review, with reasons for exclusions at each stage, ideally with a flow diagram | 8 |
| Study characteristics | 18 | For each study, present characteristics for which data were extracted (such as study size, PICOS, follow-up period) and provide the citations | 8 |
| Risk of bias within studies | 19 | Present data on risk of bias of each study and, if available, any outcome-level assessment (see item 12) | No |
| Results of individual studies | 20 | For all outcomes considered (benefits or harms), present for each study (a) simple summary data for each intervention group and (b) effect estimates and confidence intervals, ideally with a forest plot | 8 |
| Synthesis of results | 21 | Present results of each meta-analysis done, including confidence intervals and measures of consistency | 8 |
| Risk of bias across studies | 22 | Present results of any assessment of risk of bias across studies (see item 15) | 8 |
| Additional analysis | 23 | Give results of additional analyses, if done (such as sensitivity or subgroup analyses, meta-regression [see item 16]) | 8 |
| **Discussion** |  |  |  |
| Summary of evidence | 24 | Summarise the main findings including the strength of evidence for each main outcome; consider their relevance to key groups (such as health care providers, users, and policy makers) | 10 |
| Limitations | 25 | Discuss limitations at study and outcome level (such as risk of bias), and at review level (such as incomplete retrieval of identified research, reporting bias) | 11 |
| Conclusions | 26 | Provide a general interpretation of the results in the context of other evidence, and implications for future research | 12 |
| **Funding** |  |  |  |
| Funding | 27 | Describe sources of funding for the systematic review and other support (such as supply of data) and role of funders for the systematic review | 18 |

1. Liberati A, Altman D G, Tetzlaff J, et al. The Prisma Statement for Reporting Systematic Reviews and Meta-Analyses of Studies That Evaluate Healthcare Interventions: Explanation and Elaboration. *BMJ*. 2009;339:b2700.


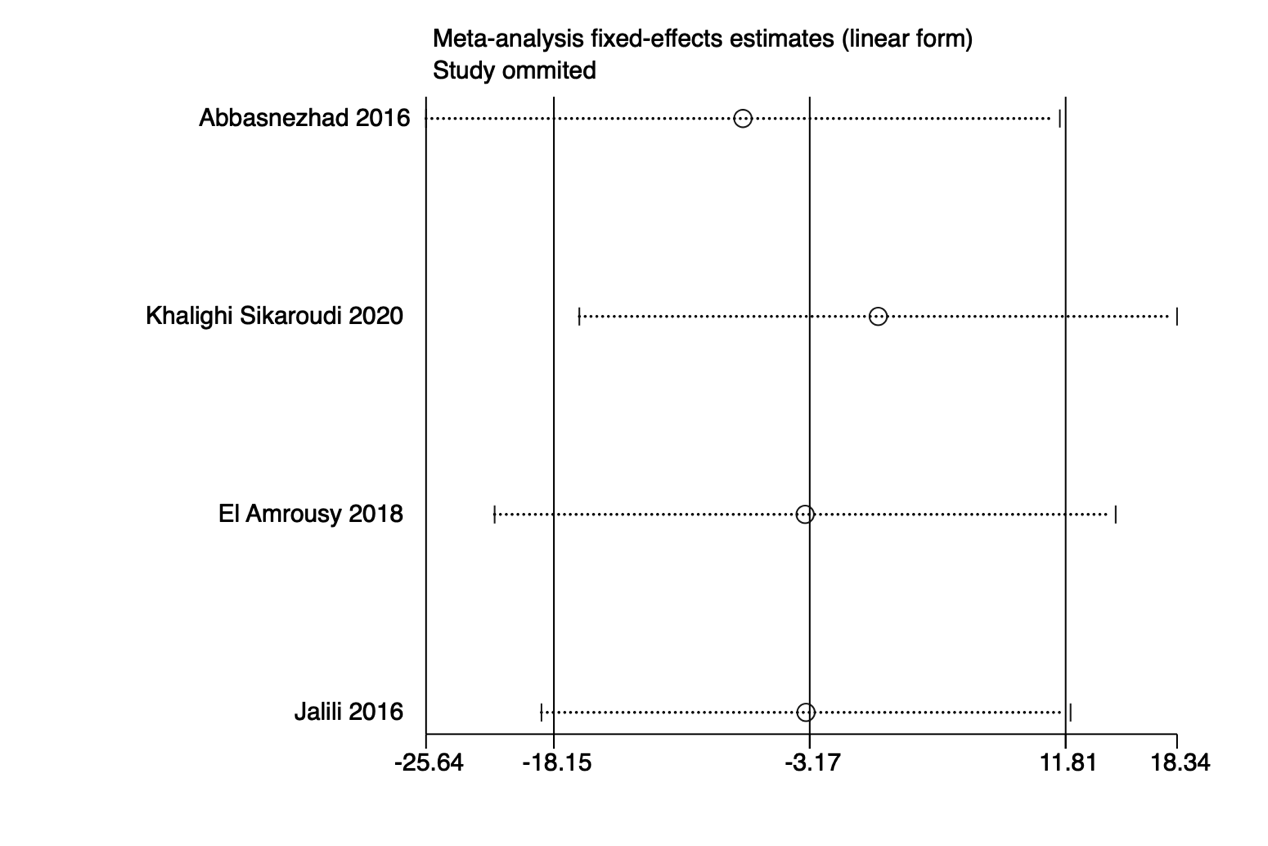


**Supplementary Figure S1.** Sensitivity analyses of comparing IBS-SSS between participants of intervention group and placebo group before intervention.


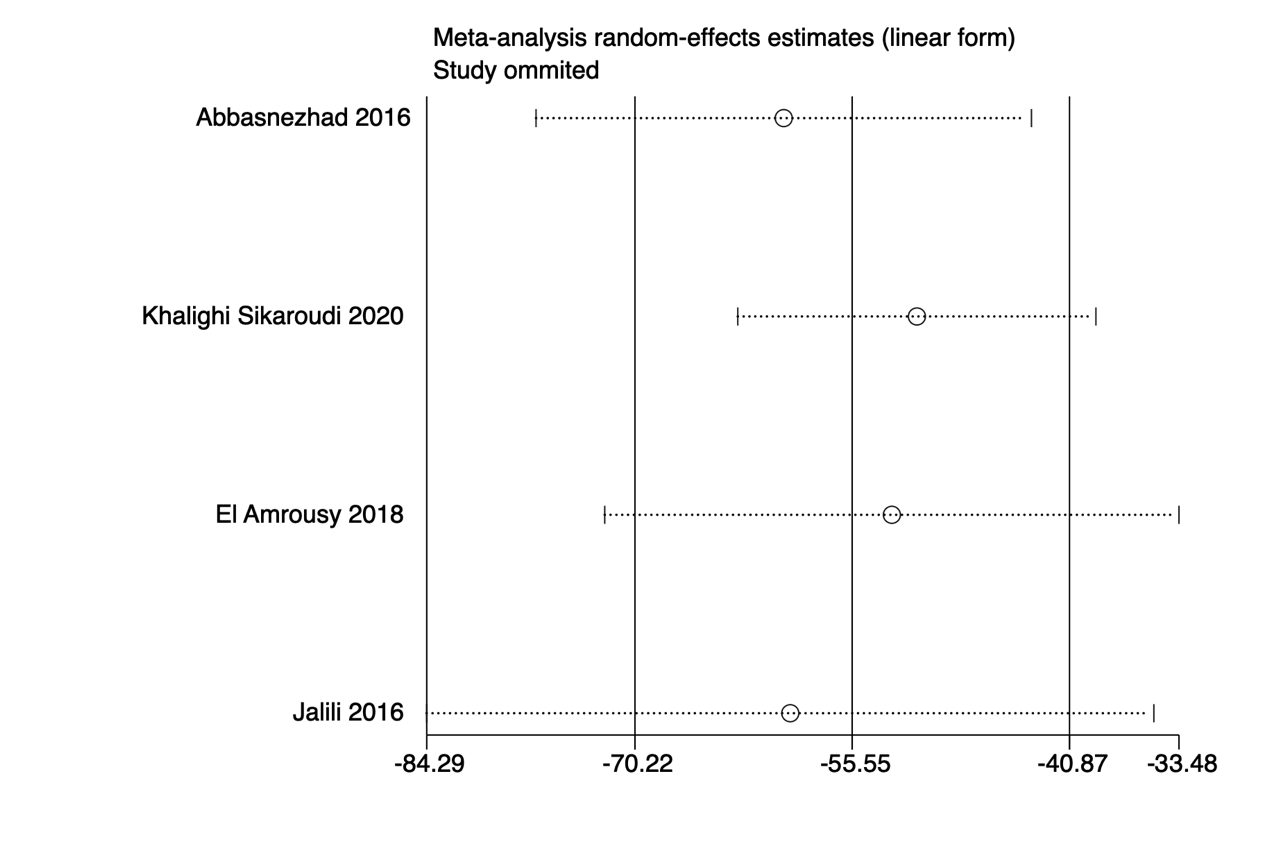


**Supplementary Figure S2.** Sensitivity analyses of comparing IBS-SSS between participants of intervention group and placebo group after intervention.


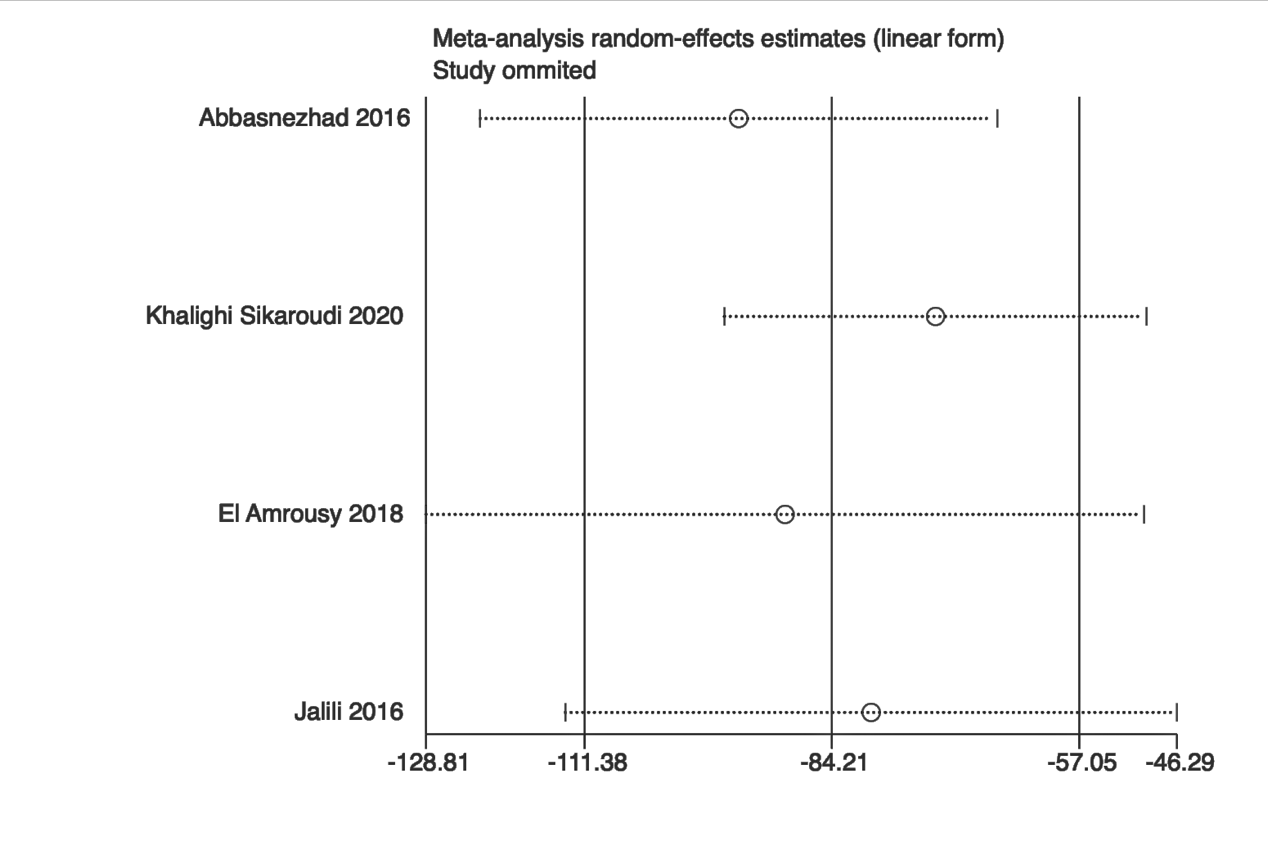


**Supplementary Figure S3.** Sensitivity analyses of the improvement of IBS-SSS after receiving vitamin D supplementation.


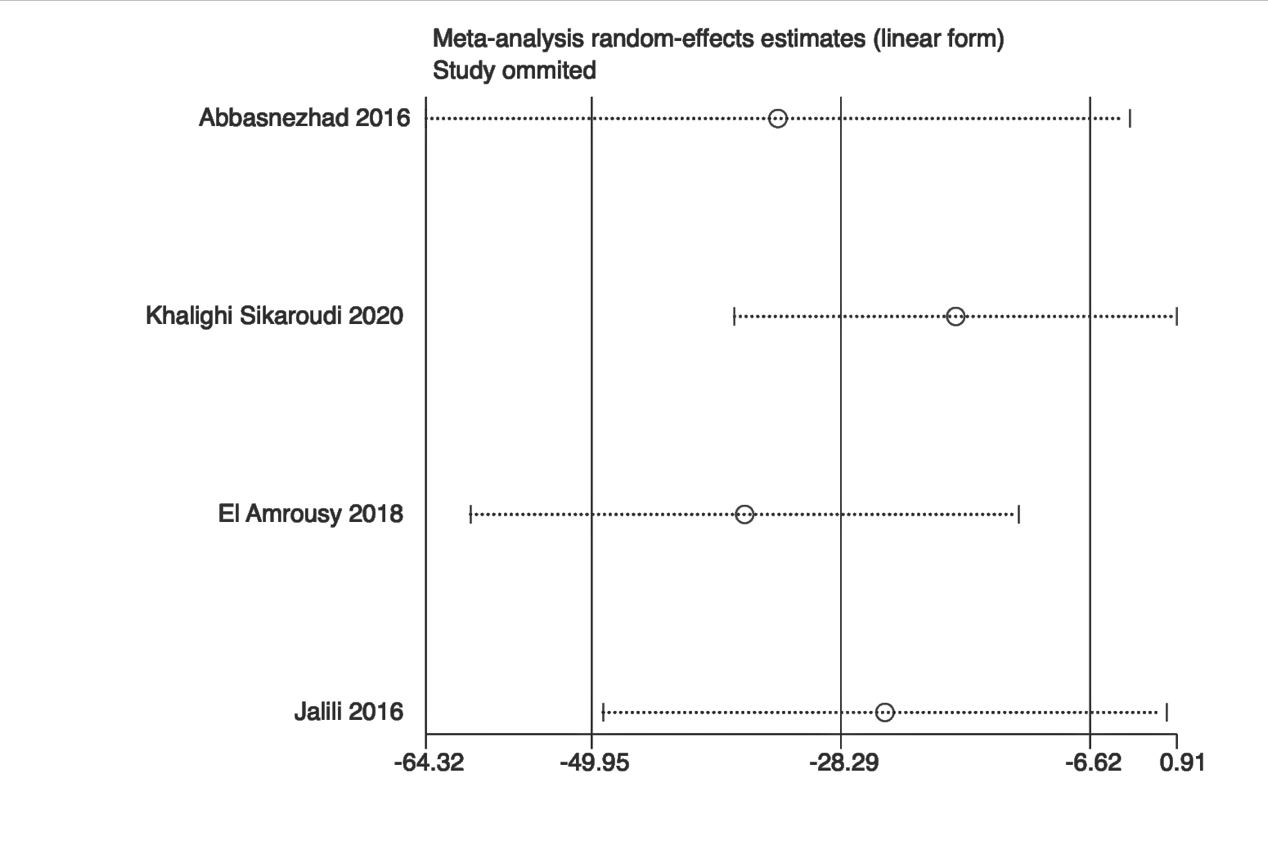


**Supplementary Figure S4.** Sensitivity analyses of the improvement of IBS-SSS after receiving placebo.


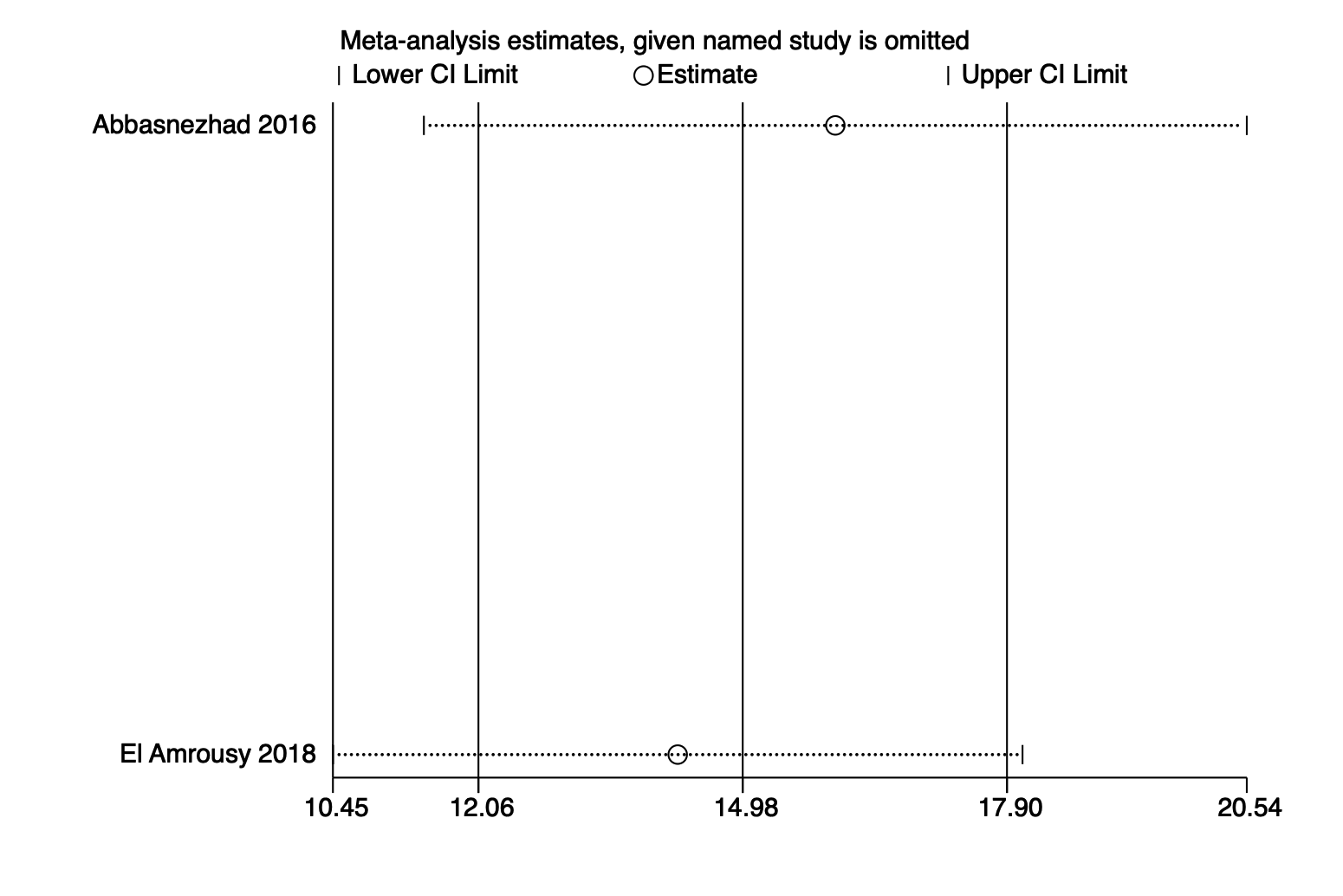


**Supplementary Figure S5.** Sensitivity analyses of the improvement of IBS-QoL after receiving vitamin D supplementation.


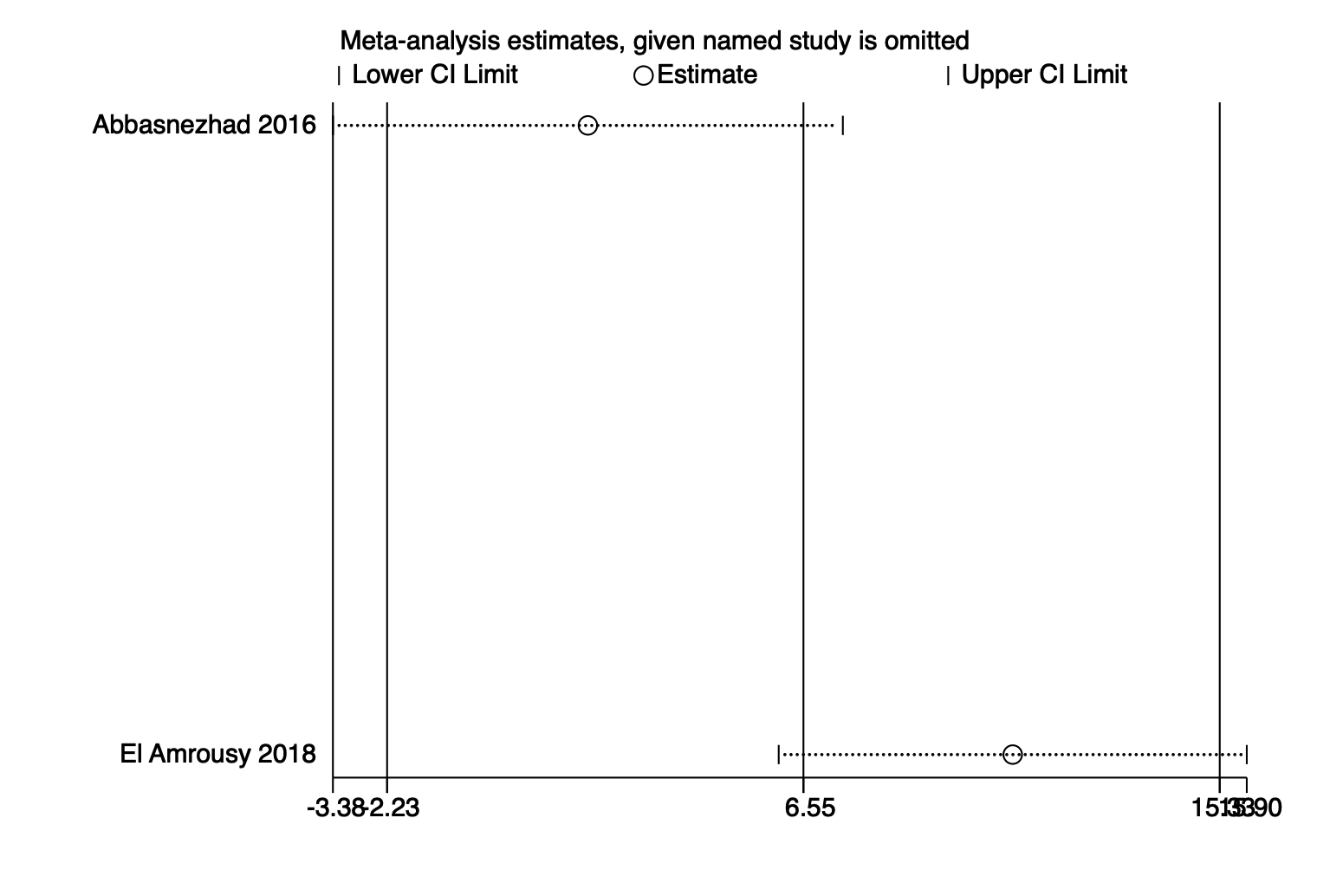


**Supplementary Figure S6.** Sensitivity analyses of the improvement of IBS-QoL after receiving placebo.
